# Phylogeny and ultrastructure of *Elthusa raynaudii* (Isopoda, Cymothoidae) firstly recorded from the invasive silver cheeked toadfish (*Lagocephalus sceleratus*) (Gmelin 1789) in eastern Mediterranean Sea coast

**DOI:** 10.1007/s00436-023-08100-1

**Published:** 2024-01-06

**Authors:** Nisreen E. Mahmoud, Magdy M. Fahmy, Marwa S. Khattab, Mai Abuowarda

**Affiliations:** 1 Department of Parasitology, Faculty of Veterinary Medicine, Cairo University, Giza, PO 12211 Egypt; 2 Department of Pathology, Faculty of Veterinary Medicine, Cairo University, Giza, PO 12211 Egypt

**Keywords:** Lessepsian pufferfish, Mediterranean, Isopods, *Elthusa raynaudii* Morphology, Molecular identification

## Abstract

With the opening of the Suez Canal in 1869, many changes have occurred in the Mediterranean Sea ecosystem so became a home to many invasive Lessepsian marine species that have migrated from the Red Sea. About 500 marine species including pufferfish have immigrated and rapidly established a population in the Mediterranean Sea causing significant impact on its ecosystem and fisheries sector. The parasitic fauna of these pufferfish has scarcely been studied in the Mediterranean Sea and also in their native habitat. During this surveillance study on the invasive pufferfish species from the Egyptian Mediterranean coast, the female cymothoid isopod *Elthusa raynaudii* was detected from the branchial cavity and also in the buccal cavity of 23.9% of the examined *Lagocephalus sceleratus*. The isolated isopod species was firstly identified and described through electron microscopy and molecular phylogeny based on the sequences of mitochondrial 16S rRNA gene. Additionally, the description of eggs, embryonic stage, and manca of *E. raynaudii* was firstly provided. The pathological impact on the infested fish tissues was investigated and revealed curling and loss of secondary gill lamellae in addition to mucous exudates in between the gill filaments and granuloma formation in the gill arch. The study provided the first report of *L. sceleratus* as a new host for the isopod *E. raynaudii* collected from the Egyptian Mediterranean coast as a new locality record. The role of the Lessepsian invasive pufferfish in transmitting parasites to the native fish species was discussed*.*

**Graphical Abstract:**

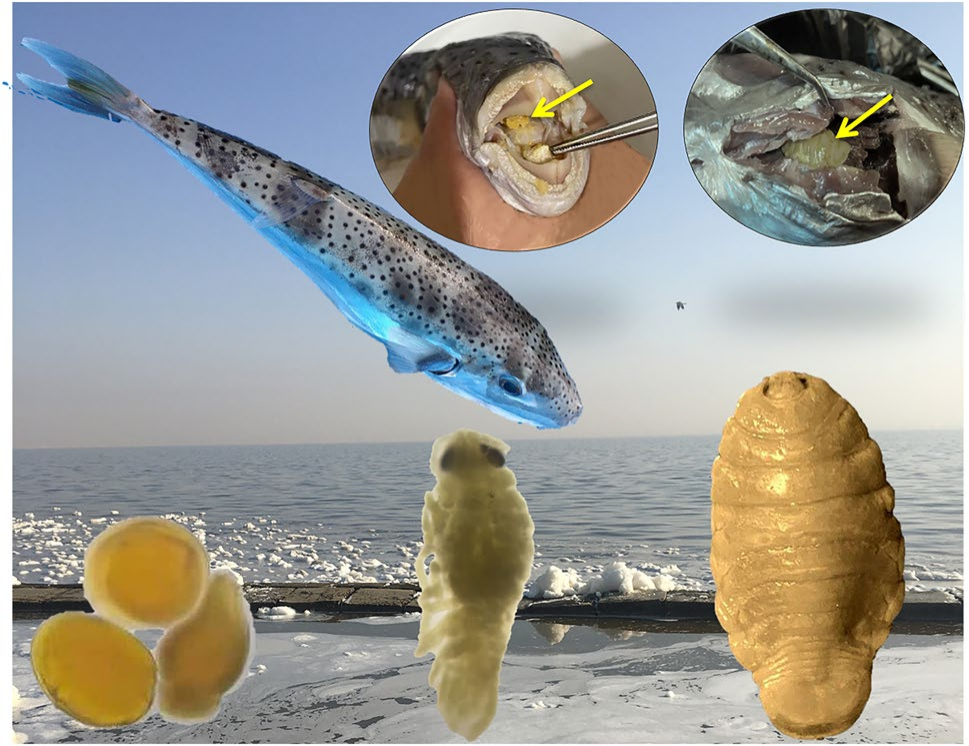

## Introduction

Breaking of natural aquatic barriers results in the spreading of some species into the ecosystems where they are alien. This dispersion can affect the native species and also the equilibrium of the aquatic invaded ecosystems (Golani 1998). Decline in the number of native species and introduction of pathogens are the negative impacts of this invasion (Oral 2010). The Mediterranean Sea became a home to many invasive marine species that have entered through many routs including the Suez Canal which is considered the major way of migrating the Lessepsian species from the Red Sea to the Mediterranean and vice versa (Bariche et al. 2004). Also, Gibraltar Strait and ship’s ballast water are important sources for the alien species introduction into the Mediterranean Sea. The silver-cheeked toadfish* Lagocephalus sceleratus* is one of the Lessepsian species which migrated through the Suez Canal and rapidly established a population in the Eastern Mediterranean (Yaglioglu, 2011). This Lessepsian species is considered to be among the worst invasive species in the Mediterranean Sea due to its significant impact on the aquatic ecosystem and fisheries sector (Streftaris and Zenetos 2006; Ozturk 2010). Migration of Lessepsian species has been increased on the Egyptian Mediterranean coast year after one and many cases of human death after consumption of pufferfish containing Tetrodotoxin poison were reported (Farrag et al. 2016) [7]. Cymothoidae (Crustacea, Isopoda) is among the most diverse parasitic isopods infesting various fish hosts. They are attached to the host body surface, buccal, or gill cavities (Mahmoud et al. 2014a, b; Trilles and Justine 2010) and occasionally burrowing in the musculature of their hosts, causing sever tissue damage and fish mortalities (Rhode 2005; Smit et al. 2014). In Egypt, interest on the Mediterranean isopods has increased in the last decade; it was reported as the main source of isopod infestation problems occurred in marine fish farms and the inland lakes. In 2013, different species of cymothoid isopod were transported to Qarun Lake with the infected wild mugiliid fry from the Mediterranean Sea resulting in complete destruction of the lake fish stock (Mahmoud et al. 2019a and 2023). Research on the pufferfish was restricted and mostly focused on its habitat (Kalogirou et al. 2012) and impacts on fish and fisheries (Kalogirou 2013), reproduction (Rousou et al. 2014) and consumption safety issues (Deeds et al. 2008; Katikou et al. 2009). Four studies were conducted on pufferfish parasitism from the Suez Canal and Mediterranean Sea with no record of isopod infestation (El-Lamie and Abdel-Mawla 2012; Ozak et al. 2012; Bakopoulos et al. 2017; Gabel et al. 2022). As data concerning parasitic isopod on pufferfishes is scarce in the eastern Mediterranean, the objective of this study was to identify the parasitic isopod of migrant *L. sceleratus* and estimate its role in transmitting parasitic isopod species to the invaded Egyptian Mediterranean sea coast. Additionally, the pathological impact of the identified isopod species on the affected fish tissues was illustrated and discussed.

## Materials and methods

### Sample collection and study area

**Fig. 1:**
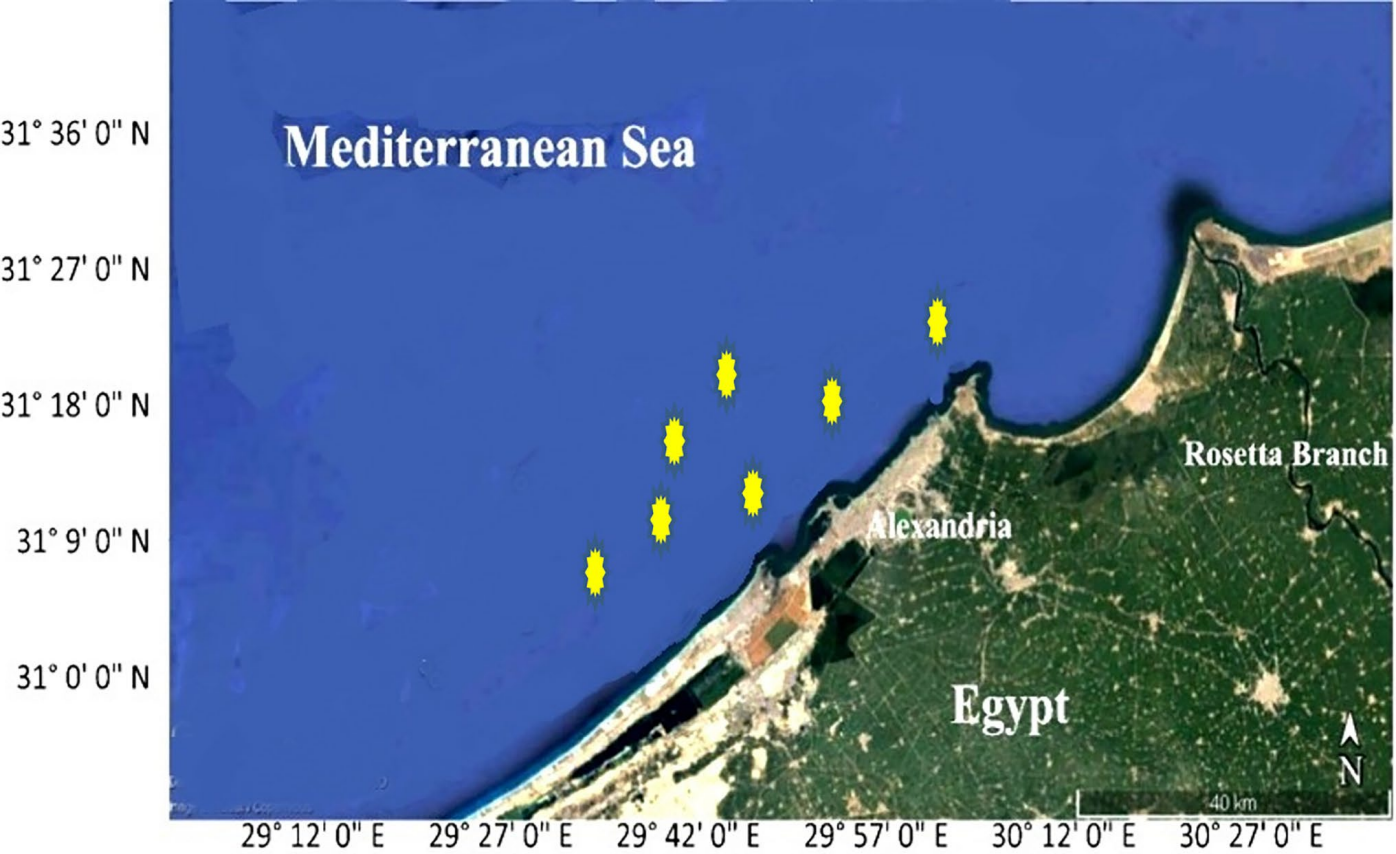
Map of the Egyptian coast of Mediterranean Sea, Alexandria (Sampling area). Marked point of sampling collection. Map was carried out using ArcGIS (ArcMap) version 10.1 software

**Fig. 2:**
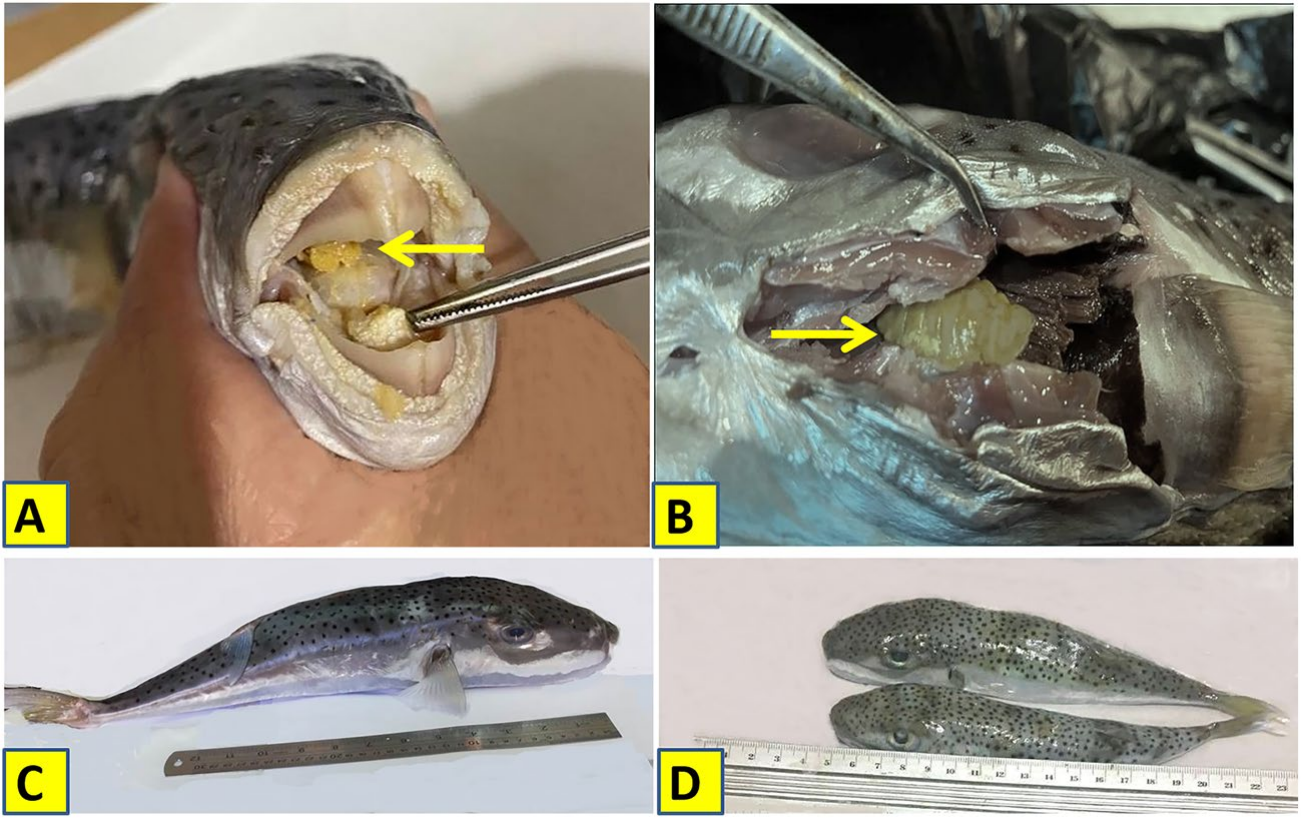
**A** *Elthusa raynaudii* female at the base of the buccal cavity* L. sceleratus*. **B** *E. raynaudii* attached to the gills (arrows).** C, D** *L. sceleratus* fish length

A total of 134 specimens of freshly dead *Lagocephalus sceleratus* (Gmelin,1789) were collected from local fishermen along the Egyptian coast of Mediterranean Sea, Alexandria (31° 00′ and 31° 36′ N and 29° 18′ and 30° 05′ E) (Fig. 1) from December to May 2022. Fish samples (15–47 mm in length) (Fig. 2C, D) were collected using commercial trawlers at a depth of 20–40 m and at a distance of about 2–3 miles from the coast. Fish samples were macroscopically inspected on spot, and each sample was placed in a bag with ice and transported to the laboratory of parasitology, Faculty of Veterinary Medicine, Cairo University, for further examination. Interviews with the local fishermen along the study area revealed that this fish species is being caught as by-catch in significant numbers and attacks fishes captured in the nets and also damages the fishing gears using its strong teeth.

### Parasitological examination

The fish body surface and all openings (skin, fins, gills, eyes, nostrils, anus, and buccal and branchial cavities) were macroscopically investigated. The detected isopods were gently removed from the infested fish. Eggs were obtained from the ovigerous females by cutting the marsupium using a dissecting needle and fine forceps (Hadfield and Smit 2020). Manca larvae released from some of the live ovigerous females were also collected. The embryonic stage was recovered from the eggs after cutting the egg envelope and removing the vitelline membrane. The parasite specimens were counted, accurately measured using an ocular micrometer, and stored in 70% ethyl alcohol. The investigated fish species was identified as *Lagocephalus sceleratus* according to Otero et al. (2013) and Farrag et al. (2016). Prevalence and intensity of infestation were calculated following Margolis et al. (1982).

### Morphological examinations and taxonomy of the isolated isopods

The freshly dead and the preserved isopod specimens were light microscopy examined using a dissecting stereo-microscope (Olympus Japan SZ40) and photographed by a digital camera (Canon 12 megapixel). The morphological identification and taxonomic classification of the isolated isopods were performed according to the keys of Bruce (1990) and Van der Wal (2019).

### Morph-metric ultrastructure examination

The isopod specimens were washed with 0.9% physiological saline solution, fixed in 2.5% glutaraldehyde (Colwell et al. 2007), dehydrated using an ethanol series (95% and 100%) for 10 min, then subjected to critical point drying in a CO_2_ drier (Autosamdri-815, Germany) (Lee 1992). The specimens were then fixed over SEM stubs, coated with gold (Spi-Module Sputter Coater, UK), and examined and photographed by SEM (JSM 5200, Electron Probe Microanalyzer, Jeol, Japan) in the Electron Microscope Unit at the Faculty of Agriculture, Cairo University.

### Molecular identification

#### DNA extraction and amplification of 16S rRNA

DNA from the isolated isopod species were extracted using DNeasy Tissue Kit (Qiagen, Germany) according to the manufacturer’s instructions. The extracted DNAs were stored at − 20 °C till used. The primers, Fish-F1 (5′AGCC-CTGTTCAATGGGATTA-3′) and Fish-R1 (5′TCCCTGGGGTAGTTTCATCTT-3′), were used with the extracted DNAs to amplify a 532 bp fragment of the 16S ribosomal RNA gene (Thangara et al. 2014). PCR reaction was done according to Thangara et al. (2014) and Mahmoud et al. (2019b).

#### DNA sequencing and phylogenetic analysis

The positive specimens of PCR products were purified by using a QIA quick purification kit (Qiagen, Germany), then sequencing using Big Dye Terminator V3.1 kit in an ABI 3500 Genetic Analyzer (Applied Biosystems, USA). The detected sequences were compared with those available in the GenBank using a BLAST server on the NCBI website (Ali et al. 2021).

#### Nucleotide sequence

Partial sequences of the isopod species 16S rRNA gene were submitted to GenBank. Sequences were aligned against other sequences of the 16S rRNA gene recorded worldwide. The analysis was carried out using the Clustal W, BioEdit software (ver. 7.0.9). A Maximum Likelihood method and Tamura-Nei model were done for phylogenetic tree using Mega 6.06 software, and bootstrap analysis was obtained with 1000 replicates (Hegab et al. 2022a, b). Pairwise comparisons were constructed using K2P model distance within *Elthusa raynaudii* compared with the most similar reference sequences (GenBank) (Mega 6.06 software) and detect inter- and intra-species variations of genetic distance values of isolated isopods (Thangara et al. 2014).

#### Nucleotide sequence accession number

Partial sequences of *Elthusa raynaudii* (Milne-Edwards 1840) adult stages isolated from* Lagocephalus sceleratus* (Rabbit fish) in Egyptian Mediterranean Coast. 16S rRNA gene was submitted to GenBank, with accession numbers ON599340.1 and OK316895.1, respectively.

### Histopathological examination

Infested gill tissues with the attached isopods were fixed in 10% neutral buffered formalin, dehydrated in ascending grades of ethanol, cleared in xylene, and embedded in paraffin. 3-µm-thick tissue sections were made using a microtome (Leica 2135, Germany) and stained with hematoxylin and eosin stain, Giemsa stain, and periodic acid stain (PAS) (Suvarna et al. 2012). A light microscope equipped with a digital camera was used for examination and capturing photographs.

## Results

### Prevalence of isopod infestation

Out of the examined 134 *L. sceleratus* samples, 32 were found infested with the isopod *Elthusa raynaudii* (23.9%). The total number of the detected parasites was 41 with a mean intensity of 1.29 ± 0.46.

### Attachment mode

All the detected female *Elthusa raynaudii* were found in *L. sceleratus* branchial cavities, attached to the gills, and only one case showed female at the base of the buccal cavity (Fig. 2A, B).

### Taxonomy and morphometric description of the isolated isopod

#### Taxonomy

The recovered isopod species was identified as *Elthusa raynaudii* (Milne-Edwards 1840) (order: Isopoda, suborder: Cymothoida, superfamily: Cymothoidea, family: Cymothoidae, genus: *Elthusa*, and species: *Elthusa raynaudii*).

### Morphometric description of the isolated isopod (light stereo-microscopy)

#### Female Elthusa raynaudii (Milne-Edwards 1840)

**Fig. 3:**
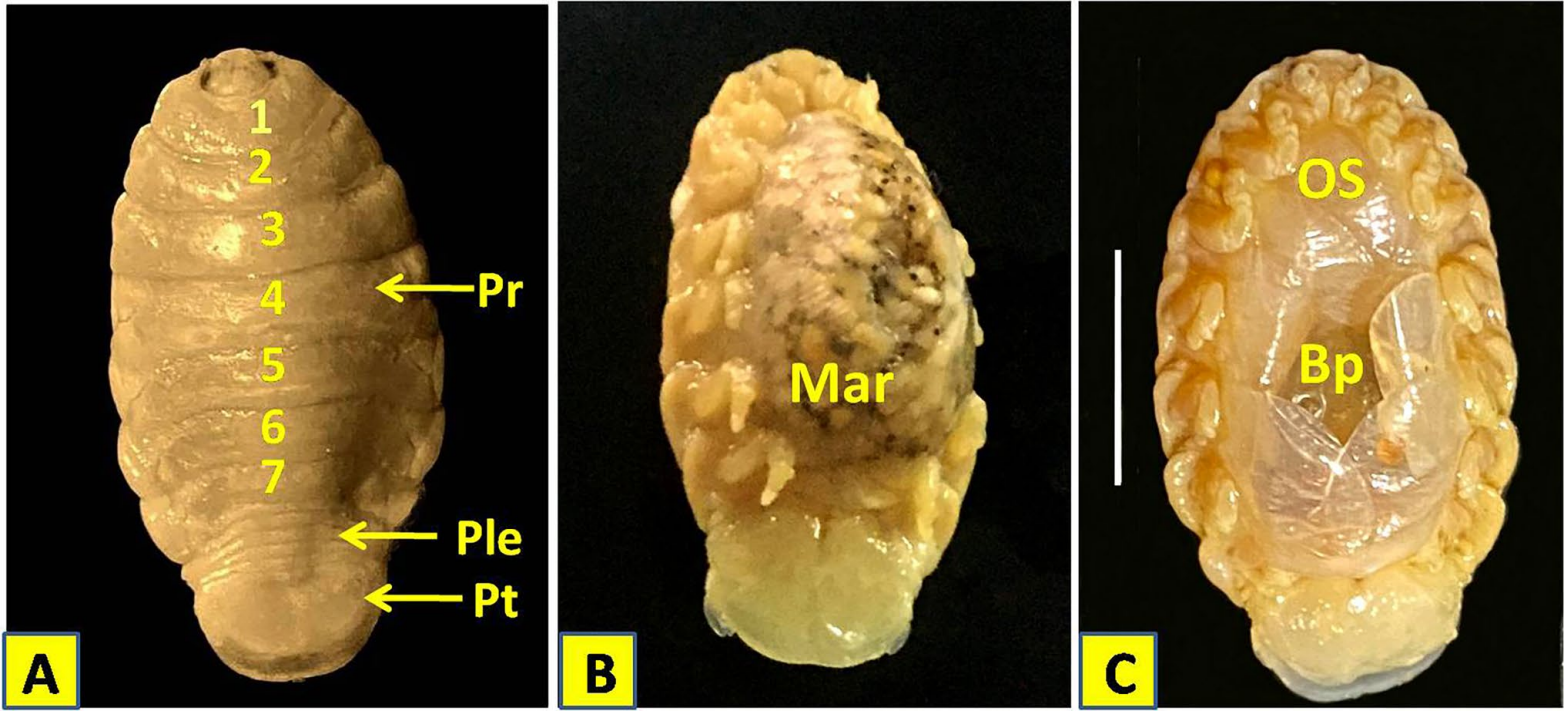
Body parts of the isolated female *Elthusa raynaudii*. **A** Dorsal view of female. **B** Ventral view of ovigeorus female. **C** Ventral view of female Showed brood pouch of 5 pairs of alternately overlapping oostegites. Scale bar (S.b) = 10 mm. Pr: perionite; Ple: pleonite; Pt: pleotelson; Mar: marsupium; Os: oostigite; Bp: broad pouch

**Fig. 4:**
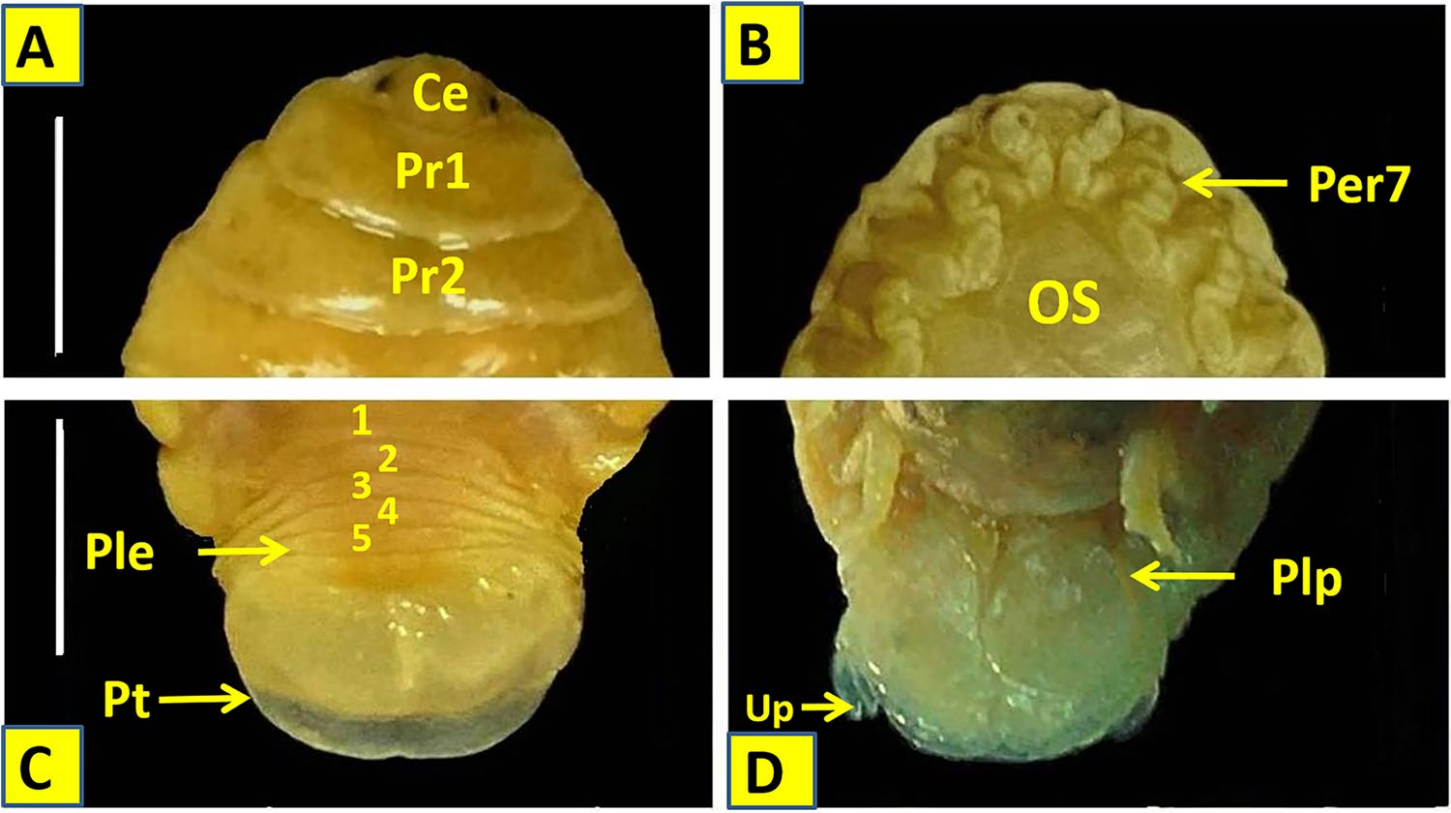
**A** Anterior part of *E. raynaudii* dorsal view. **B** Ventral view. **C** Posterior part of *E. raynaudii* dorsal view. **D** Posterior part ventral view. (S.b) = 10 mm. Ce: cephaon; Pr1: perionite 1; Pr2: perionite 2; Co: coxa; Ple: pleonite; Pt: pleotelson; Per 7: pereopod 7; Plp: pleopod; Up: uropod

The body is dark creamy in color and the dorsal surface is smooth and polished in appearance. It is ovate, measuring 23–28 (mean: 24.86 ± 1.52) mm long and 13.2–14.1 (mean: 13.60 ± 0.27) mm wide, and appears symmetrical or slightly twisted to one side in some specimens. The greatest width is at pereonite 5. The Cephalon is sub-truncate and partially immersed in pereonite 1 with blunt ventrally folded frontal margin. Eyes are oval with prominent margins. Lateral posterior margins of pereonites are moderately curved. The anterior border of pereonite 1 is straight medially and curved laterally with narrow rounded antero-lateral angle extends to the medial region of eyes. Pereonites 2–5 are subequal, and pereonites 6 and 7 are slightly narrower. Pereonite 7 has roundly pointed postero-lateral angles. Coxae 2 and 3 are wide with rounded postero-ventral angles. Coxal plates of pereonites 5–7 are conspicuous in dorsal view. Coxae 4–7 are not extended beyond the pereonite posterior margin. The pleon is short. Pleonites 1–4 posterior margins are concave while that of pleonite 5 is straight. Pleonite 1 is slightly narrower then pleonite 2 and largely concealed by pereonite 7. Pleonite 2 are partially overlapped by the postero-lateral margin of pereonite 7. Pleonites 3–5 are subequal in length, with postero-lateral angles narrowly rounded. Pleotelson is semiarc in shape with round posterior end. It is narrower than pereonite 7 and about 1.35 times as wide as long and 0.2 of the total length*.* Brood pouch is made up of 5 pairs of alternately overlapping oostegites arising from the coxae 1–4 and 6 (Figs. 3 and 4). Number of eggs per brood pouch ranged from 85 to 120 depending on the size of the ovigerous female.

## Eggs

**Fig. 5:**
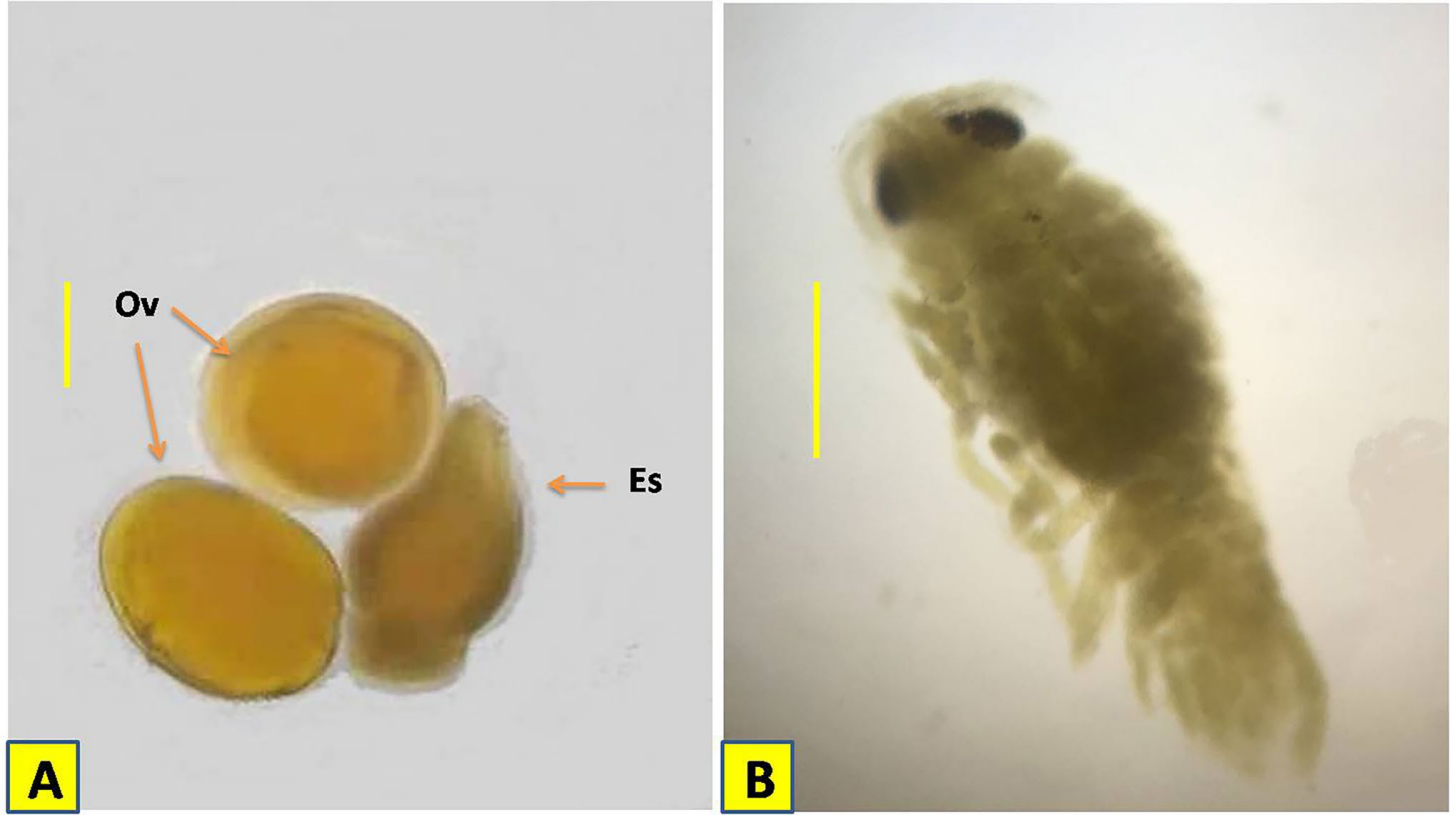
**A** Ova and embryonic stage of *Elthusa raynaudii*. S.p = 0.3 mm. **B** The manca larva (just after spawning) S.p = 0.3 mm. Ov: Ova; Es: embryonic stage

Eggs are ovoid in shape, dark yellow in color, measuring 1.01–1.03 × 0.95–0.98 mm in diameter (Fig. 5A).

## The embryonic stage

The body is between 1.18 and 1.23 mm in length and 0.62 to 0.79 mm in width (Fig. 5A).

## The manca larva (just after spawning)

The manca body length is 1.25–1.36 mm. It has 7 pereonites and 6 pairs of pereopods and obvious large compound eyes. The cephalon is semi-circular in shape. The preon consists of 7 pereonites and the pleon is narrowed with 5 pleonites progressively decrease towards the posterior. Pleotelson posterior margin is rounded with numerous setae. Antennule composed of 6 articles and shorter than antenna which composed of 11 articles and both provided with spiny setae. All pereopods similar with acute dactyli increase slightly in length from pereopod 1 to 6, with spines or setae on pereopods 2–6. Pleopods are with lamellar rami and provided with long setae. Uropod rami apices are rounded and extend beyond the posterior of pleotelson. Rami are subequal in length with plumose setae on the posterior margin (Fig. 5B).

## SEM examination of the female Elthusa raynaudii

**Fig. 6:**
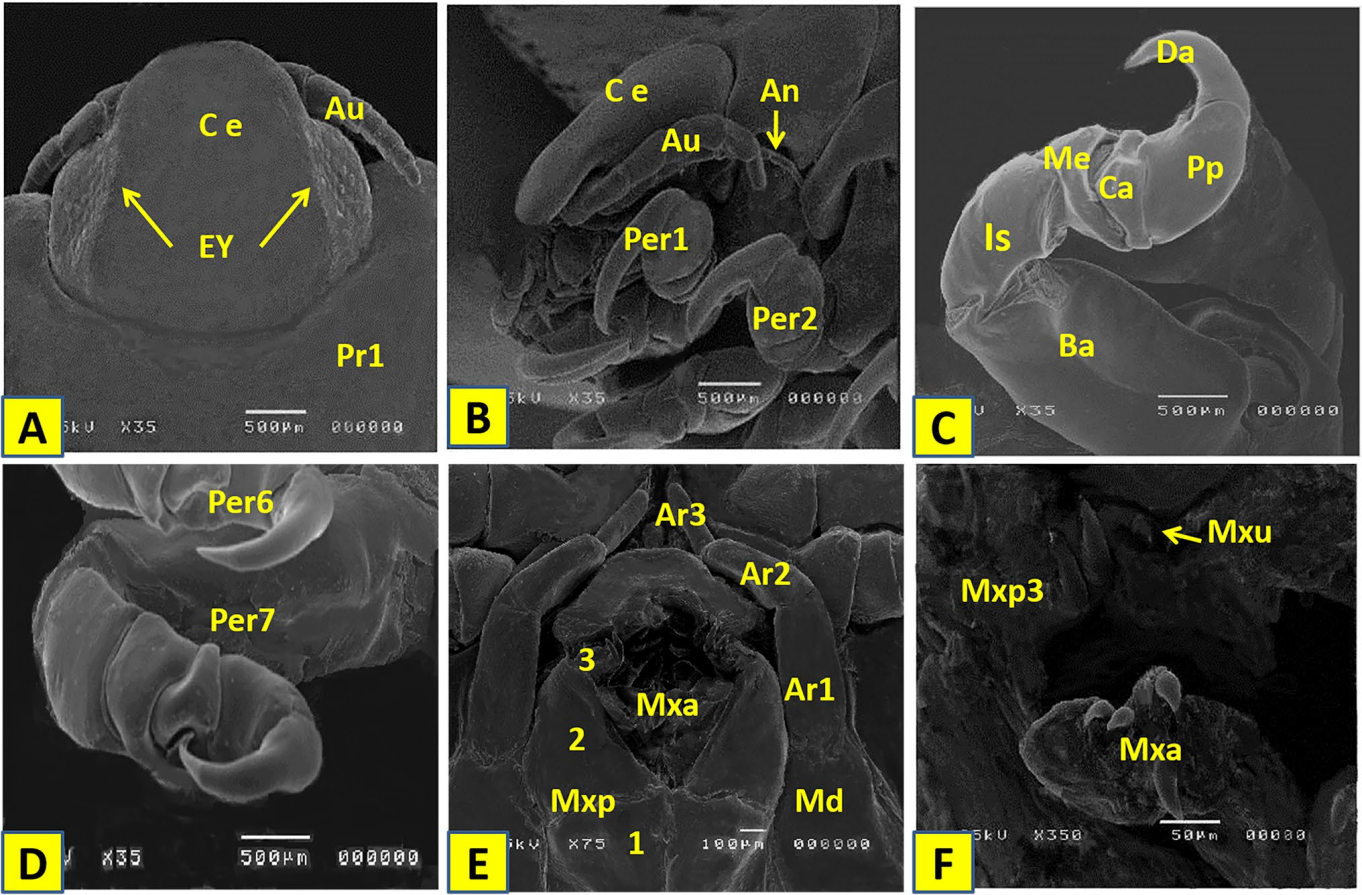
**A** Cephalon of female *Elthusa raynaudii* dorsal view. **B** Cephalon lateral view. **C** Pereopod 1. **D** Pereopod 6 and 7. **E, F** Mouth parts; An: antenna; Au; antennule; Ce: cephaon; Ey: eye; Pr1: pereonite 1; Per1: pereopod 1; Per2: pereopod 2; Da: dactylus; Pp: propodus; Ca: carpus; Me: merus; Is: ischium; Ba: basis; Md: Mandible; Ar 1: Md article1; Ar 2: Md article2; Ar 3: Md article3; Max: Maxilla; Mxp: Maxilliped; Mxp3: Maxilliped article 3; Mxu: Maxillule

**Fig. 7:**
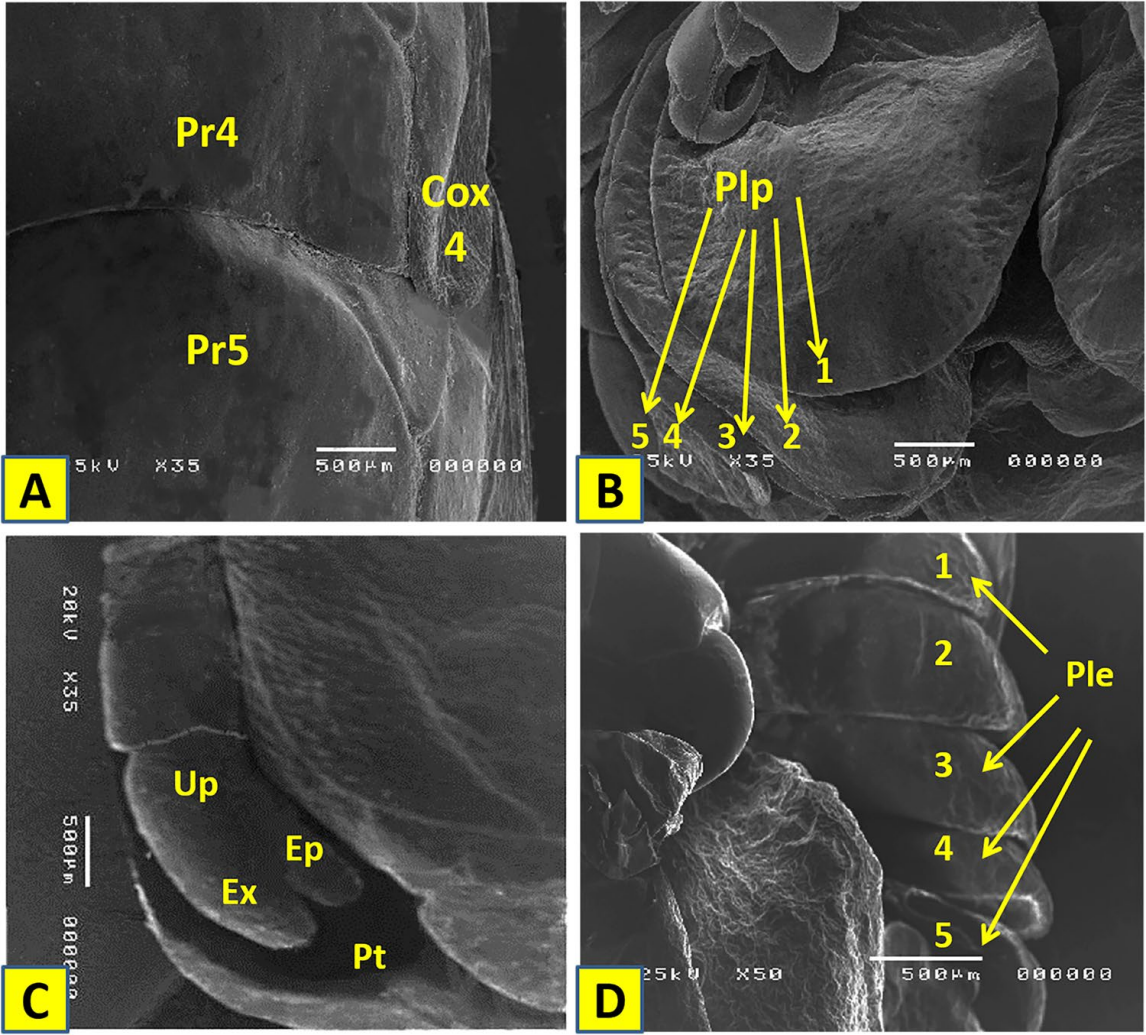
**A** Pereonites of *Elthusa raynaudii*. **B** Pleopods. **C** Uropod and pleotelson. **D** Pleonites. Pr4: pereonite 4; Pr5: pereonite 5; Cox 4: coxa 4; Plp: pleopods; Up: uropod; Ep: exopod; Ep: endopod; Pt: pleotelson; Ple; pleonite

Antennule is shorter than the antenna and composed of 8 articles. Articles are longer than wide. Article 2 is with 2 setae and a tuft of setae on the terminal article. Antenna is thinner than antennules, consists of 11 articles, and extends to about the middle of pereonite 1. It has 3 setae on both article 9 and 10 and a tuft of setae on the terminating article (Fig. 6A, B). Mandible has prominent molar. Article 2 of the mandibular palp has 5 setae, and article 3 has 2 setae. Maxillule is with 4 spines. Maxilla has 2 spines on each of medial and lateral lobes respectively. Maxilliped article 3 has 2 terminal spines (Fig. 6E, F). All pereopods are without setae. Pereopod 1 basis is 1.7 times as long as the greatest width; ischium is 0.6 times as long as basis, merus has no proximal bulbous protrusion, and carpus has rounded proximal margin; propodus 2 is times as long as wide, and dactylus is 0.8 times as long as propodus and not extended beyond the carpus. Pereopod 7 basis is with carina, ischium is 0.4 times as long as basis; merus 0.6 is times as long as ischium, carpus is 0.7 times as long as ischium, propodus is 0.9 times as long as ischium, and dactylus is 0.9 times as long as the basal width. The posterior margin of Sternite 7 has 2 submedian distal fleshy lobes (Fig. B–D). Pleopods 1–5 become markedly smaller. All pleopods are simple with lamellar rami and the exopod being larger than endopod (Fig. 7A, B, D). Uropod is broadly rounded and did not reach the posterior margin of pleotelson. Exopod is about as long as the peduncle and slightly longer than the endopod (Fig. 7C).

### Genetic and phylogenetic analysis

The sequence analysis of *Elthusa raynaudii* adult stages explained that the samples were 96.35% and 95.96%, respectively, identical with *Elthusa spp.* (LC159454.1) previously isolated from Japan. This species of *Elthusa raynaudii* isolated from *Lagocephalus sceleratus* (Rabbit fish) in Mediterranean Coast, Egypt, was recorded in the GenBank on the NCBI with accession numbers ON599340.1 and OK316895.1, respectively.

**Fig. 8:**
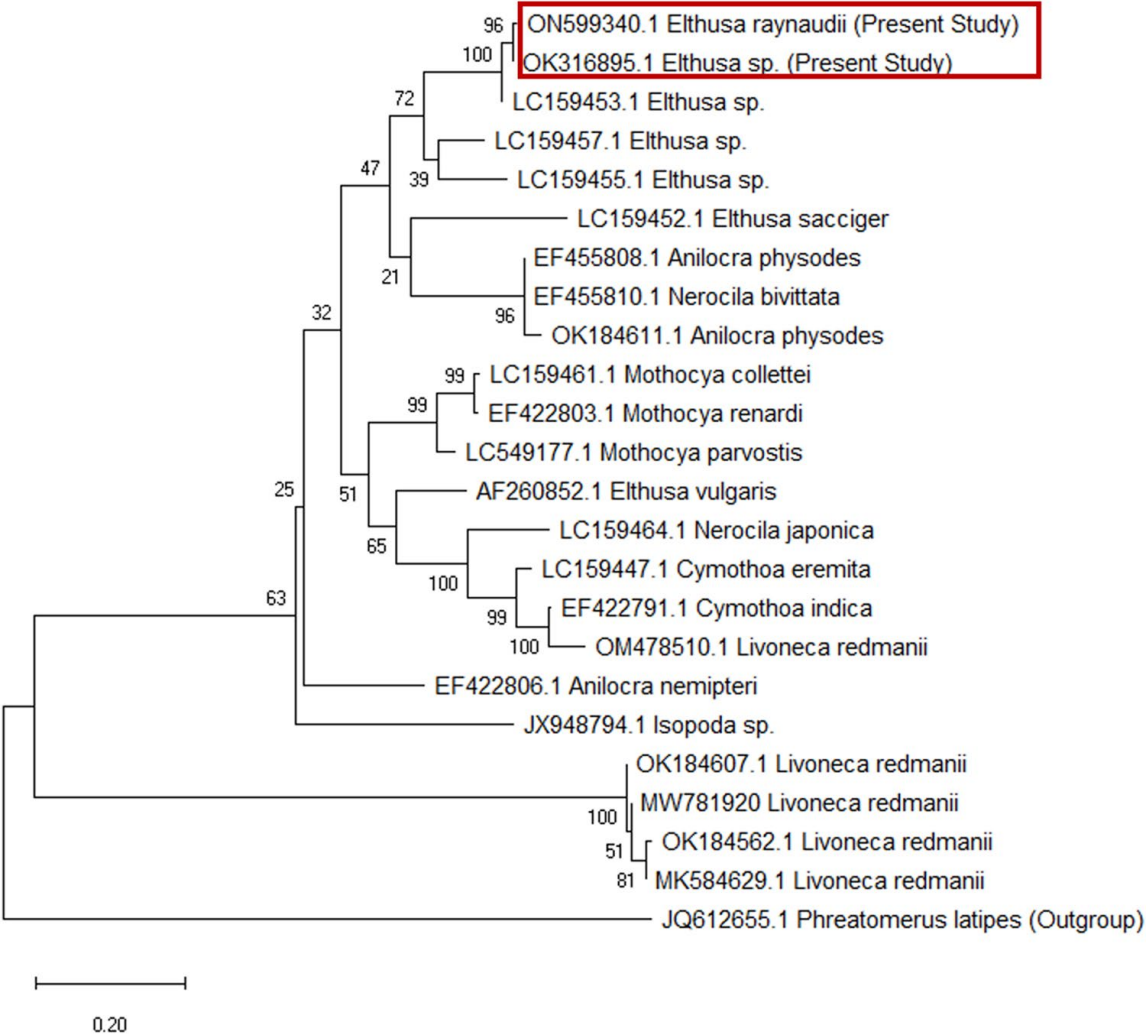
Phylogenetic relationships based on 16S ribosomal RNA (16S- rRNA) sequences of *Elthusa raynaudii* adult stages isolated from *Lagocephalus sceleratus* (Rabbit fish) in Egyptian Mediterranean Coast*.* The trees were constructed and analyzed using a Maximum Likelihood method and Tamura-Nei model. *Phreaomerus latipes* with accession number JQ612655 is considered outgroup

In this study, the PCR assays revealed an amplified DNA fragment of* Elthusa raynaudii* approximately 526–532 bp. Additionally, phylogenetic analysis based on the 16S rRNA gene data reveals that this detected isopoda was closely affiliated to the genospecies of *Elthusa* sp. isolated from Japan (LC159453.1) and can be differentiated clearly from other isopods genospecies by Maximum Likelihood method. The Maximum Likelihood method tree having 24 clades with the percentage of replicate trees in which the associated taxa clustered together in the bootstrap test (1000 replicates) (Fig. 8). The comparison between inter- and intra-species analysis of genetic distance among 24 isopod species revealed that the genetic identity of *Elthusa raynaudii* (ON599340.1 and OK316895.1) isolated from Mediterranean Coast in Egypt was verified with a high sequence homology with *Elthusa* sp. isolated from Japan (LC159453.1) (Table 1).

**Table 1:** Pairwise comparisons based on K2P model distance within *Elthusa raynaudii* compared with the most similar reference sequences (GenBank)

No	Species	1	2	3	4	5	6	7	8	9	10	11	12	13	14	15	16	17	18	19	20	21	22	23
1	JQ612655.1 *Phreatomerus latipes* (outgroup)																							
2	ON599340.1 *Elthusa raynaudii*	1.48																						
3	OK316895.1 *Elthusa* sp.	1.48	0.01																					
4	LC159453.1 *Elthusa* sp.	1.48	0.02	0.02																				
5	LC159457.1 *Elthusa* sp.	1.39	0.20	0.20	0.18																			
6	LC159455.1 *Elthusa* sp.	1.33	0.23	0.22	0.20	0.16																		
7	LC159461.1 *Mothocya collettei*	1.22	0.27	0.26	0.26	0.26	0.28																	
8	LC159452.1 *Elthusa sacciger*	1.64	0.33	0.33	0.31	0.31	0.33	0.37																
9	AF260852.1 *Elthusa vulgaris*	1.50	0.34	0.33	0.30	0.33	0.32	0.29	0.42															
10	EF422803.1 *Mothocya renardi*	1.29	0.30	0.29	0.29	0.26	0.29	0.02	0.37	0.28														
11	LC159464.1 *Nerocila japonica*	1.57	0.38	0.38	0.37	0.37	0.36	0.30	0.42	0.28	0.30													
12	EF422806.1 *Anilocra nemipteri*	1.26	0.38	0.38	0.38	0.33	0.32	0.31	0.40	0.33	0.32	0.37												
13	EF455810.1 *Nerocila bivittata*	1.33	0.31	0.31	0.31	0.25	0.30	0.29	0.41	0.33	0.28	0.41	0.35											
14	EF455808.1 *Anilocra physodes*	1.37	0.31	0.31	0.31	0.26	0.30	0.30	0.41	0.33	0.29	0.40	0.35	0.0										
15	OK184611.1 *Anilocra physodes*	1.35	0.33	0.33	0.33	0.28	0.33	0.33	0.45	0.35	0.32	0.44	0.37	0.02	0.02									
16	LC549177.1 *Mothocya parvostis*	1.39	0.34	0.34	0.34	0.27	0.29	0.09	0.40	0.24	0.08	0.30	0.30	0.27	0.28	0.30								
17	LC159447.1 *Cymothoa eremita*	1.63	0.42	0.41	0.39	0.36	0.33	0.30	0.39	0.25	0.29	0.19	0.32	0.43	0.42	0.44	0.28							
18	EF422791.1 *Cymothoa indica*	1.69	0.45	0.44	0.42	0.38	0.36	0.32	0.42	0.27	0.30	0.20	0.34	0.41	0.4	0.41	0.28	0.07						
19	OM478510.1 *Livoneca redmanii*	1.68	0.47	0.47	0.46	0.42	0.42	0.34	0.50	0.31	0.37	0.26	0.39	0.41	0.4	0.42	0.35	0.10	0.05					
20	OK184607.1 *Livoneca redmanii*	1.48	1.27	1.27	1.25	1.16	1.14	1.26	1.56	1.24	1.28	1.30	1.26	1.14	1.15	1.14	1.19	1.30	1.36	1.35				
21	OK184562.1 *Livoneca redmanii*	1.63	1.30	1.31	1.28	1.16	1.15	1.25	1.66	1.23	1.27	1.32	1.25	1.13	1.13	1.14	1.18	1.31	1.37	1.36	0.03			
22	MW781920 *Livoneca redmanii*	1.52	1.29	1.29	1.27	1.17	1.15	1.26	1.58	1.25	1.28	1.31	1.25	1.15	1.16	1.17	1.19	1.32	1.39	1.38	0.01	0.03		
23	MK584629.1 *Livoneca redmanii*	1.59	1.30	1.31	1.28	1.17	1.16	1.26	1.63	1.24	1.29	1.31	1.26	1.16	1.17	1.17	1.20	1.31	1.37	1.36	0.03	0.01	0.02	
24	JX948794.1 *Isopoda* sp.	1.41	0.53	0.52	0.49	0.46	0.40	0.45	0.49	0.43	0.45	0.51	0.45	0.49	0.49	0.51	0.48	0.45	0.48	0.52	1.39	1.40	1.41	1.4

On the other hand, low level of genetic divergence (GD) of the isolated *Elthusa raynaudii* from Mediterranean Coast, Egypt, with the genospecies of *Elthusa* sp. isolated from Japan (LC159453.1) was 0.02 detected (Table 1).

### Histopathological findings

**Fig. 9:**
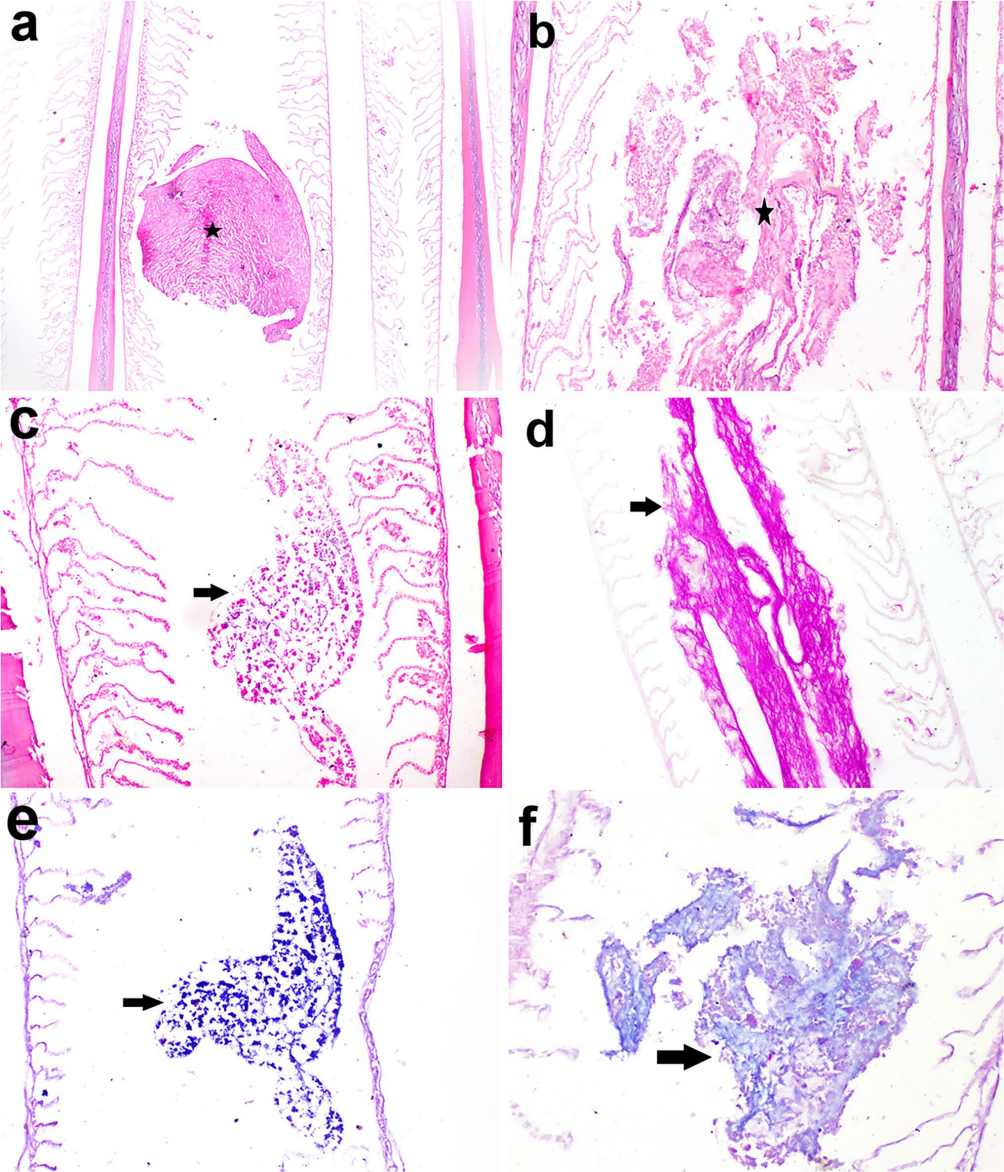
Micrographs of rabbitfish infested with isopod in the gill chamber. (**a**) Part of the parasite presents between gill lamellae (star) causing curling and pressure on secondary gill lamellae (× 40). (**b**) Occurrence of exudates between gill lamellae with complete loss of secondary gill lamellae (× 100). (**c**) Desquamated cells between gill lamellae (arrow) with curling of secondary gill lamellae (× 100). Hematoxylin and eosin stain. (**d**) PAS-positive mucous exudates in between the gill lamellae (periodic acid Schiff × 100), (**e**) dark blue-stained cells with Giemsa stain between gill lamellae (Giemsa stain × 100). (**f**) Purplish-blue organisms in the mucous exudates (Giemsa stain × 200)

**Fig. 10:**
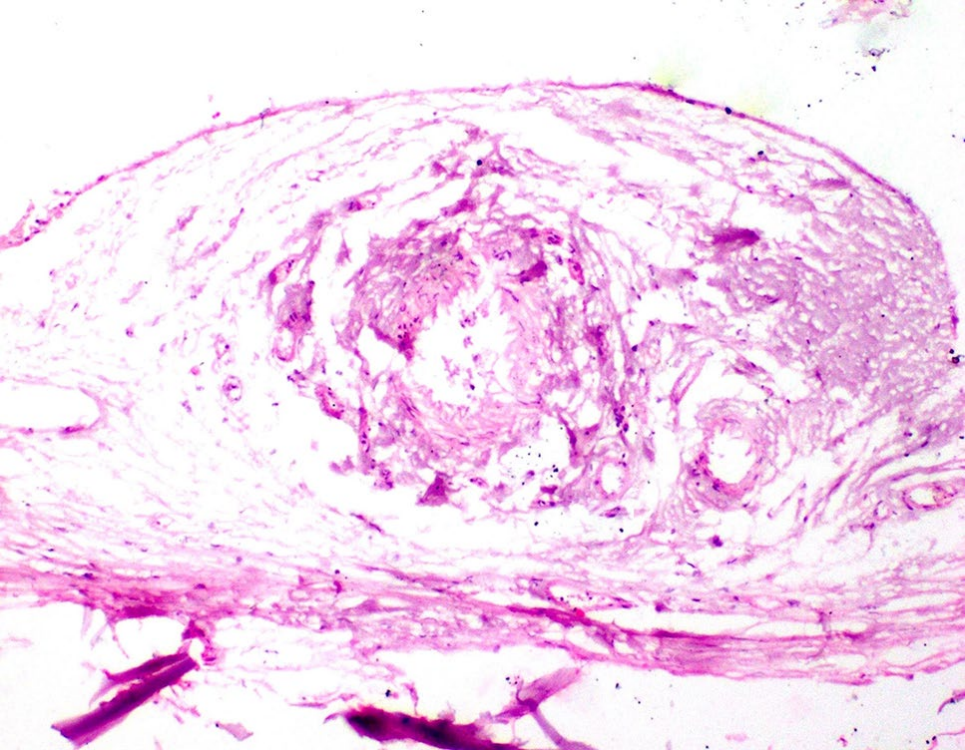
Micrographs of rabbitfish infested with isopod in the gill chamber showing granulome formation surrounded by fine rim of connective tissue. Hematoxylin and eosin stain

The parasite caused a variety of histopathological alterations in the gills of infected fish. Parts of the parasite were observed between the gill filaments exerting pressure atrophy on them. The secondary gill lamellae suffered from severe curling, erosions, and epithelial desquamation and sometimes were completely lost. Edema was also observed in the primary gill lamellae. Mucous exudates with suspected bacterial aggregations as demonstrated by PAS stain and Giemsa stain were seen in between the gill filaments. Dark blue-stained cells with Giemsa stain between gill lamellae were recorded (Fig. 9). Microscopy of the gill arch revealed a granulome which is consisted of central necrosed area and leukocytes surrounded by a fine rim of fibroblasts. The lesion caused thickening of the gill arches (Fig. 10).

## Discussion

In this study, the female cymothoid isopod *Elthusa raynaudii* was recorded for the first time from the invasive pufferfish *L. sceleratus* collected from the Egyptian Mediterranean Sea coast. The infestation rate was 23.9% found markedly higher than that reported for the isopod *Gnathia* sp. praniza larva by Bakopoulos et al. 2017 (2.4%) from the same fish species caught from northern and southern locations of the eastern Aegean Sea. Morphologically, *Elthusa raynaudii* is characterized by the cephalon with a narrow truncate rostrum; the pereonite 1 with straight anterior margin; the subequal pleonites; and the broad rounded uropod apices which extend to more than half the pleotelson length. The morphological traits and morphometric measures of the detected *E. raynaudii* were consistent with the description of Bruce (1990) and also Van der Wal et al. (2019) who collected *E. raynaudii* specimens from the west coast of South Africa in 2010 without mentioning the host Bruce (1990) and Van der Wal et al. (2019). The current study is considered the 1st provided the molecular characterization of *L. raynaudii* and recorded this isopod species in the GenBank. All the specimens of *E. raynaudii* in this study were isolated from the branchial cavity attached to the gills, but a non-ovigerous female was oddly detected in the mouth of *L. sceleratus* specimens with its cephalon directed up-side-down. The isopod in this odd location probably was either introduced to the mouth of *L. sceleratus* while preying on its natural host or the parasite migrated from the gill cavity to the mouth after death of the captured* L. sceleratus*. The same odd cases for this isopod species were reported by Hurley (1961) (in New Zealand) and Williams et al. (2010) (in Taiwan) from the stomach and the roof of the mouth of shark, respectively, and they described the cases as being accidental. The isopod *L. raynaudii* was originally described from the Cape of Good Hope in South Africa (Milne-Edwards 1840) from unknown host and also recorded several times from a wide range of localities within the Indo-Pacific region and sub-Saharan Africa (Van der Wal et al. 2019). Regarding the establishment of *L. sceleratus* in this investigated area of the Mediterranean Sea coast, it might be attributed to the high rate of water pollution recorded through our previous investigation on the water quality of this area revealing severe water deterioration and concluded a strong positive relationship between isopod prevalence and water pollution. The same conclusion was provided by Ashmawy et al. (2018) and Chapman et al. (2015). In the present study, the record of isopod infestation among the invasive pufferfish *L. sceleratus* from the Egyptian Mediterranean Sea coast indicated the role of this invasive fish in transmitting parasites such as isopods and perhaps other types of pathogens to the Egyptian marine water resources and consequently to the native fish species (Mahmoud et al. 2023). This explain the spreading of isopod fauna to the inland fish farms and lakes as occurred in Qarun Lake where many isopod species were transported with the fry from the Mediterranean Sea causing great problems (Fahmy et al. 2021). Histopathological alteration of the gills due to isopod infestation was reported in several studies (Ravichandran et al. 2010a; Kumar et al. 2012). In the current study, The recorded histopathological changes could be attributed to the pressure of the large sized isopod *L. raynaudii *inhabits the branchial cavity. The imprint of the parasite parts on the gill filament was observed associated with severe curling, erosions, and complete loss of secondary gill lamellae in severe cases. Granulome formation in the gill arches recorded in this study is similar to the report of Rameshkumar and Ravichandran (2014) and Panakkool-Thamban et al. (2016) who mentioned that the large size of the parasitic isopods causes a variety of histopathological alterations to gills including erosions, thickening of gill filaments in addition to granulomas formation. They also reported that granulomes are formed of macrophages and epithelioid cells which sometimes enclosed by a fine capsule. They also observed a lipofibrosis in which acidophilic plasmocytes, lymphocytes, and granulocytes were seen. This tissue reaction was proposed to be due to the continuous irritation of the parasite’s body and appendages and also to the blood feeding habit of the parasite (Rameshkumar and Ravichandran 2014; and Panakkool-Thamba et al., 2016). The detected bacterial aggregations in the gill lamellae of a cymothoid isopod infested host are possibly due to lesions and contamination with the respiratory water. This reduces the respiration and nitrogenous waste excretion of the host (Trilles 1994; Ravichandran et al. 2009 and Ravichandran et al. 2011). The parasite also increases the susceptibility to secondary infections with bacteria and fungi due to tissue damage, anemia, and stress (Rameshkumar and Ravichandran 2014; Purivirojkul and Songsuk 2020). Hyperplastic and hypertrophic changes in the gill lamellae were not observed in the present study unlike to previous studies which showed that gill hyperplasia and hypertrophy are the main changes observed in the gills due to isopod infestation (Ravichandran et al. 2010b). This could be related to the species of isopod and infested fish. The current study provides that *L. sceleratus* in the eastern Mediterranean should be considered an important host for the low host specificity isopod parasites. The extent to which the isopod *L. raynaudii* infestation could have a negative impact on *L. sceleratus* populations needs more further investigation. On the other hand, the recorded isopod species can accumulate in the invasive *L. sceleratus* populations over time as the opportunities for the parasite to adapt to the host increase, the case which can result in Poulin and Mouillot 2003; Cornell and Hawkins 1993.

Τo our knowledge, this study constitutes the first report on parasites infesting *L. sceleratus* from the Egyptian Mediterranean Sea. Additionally, the study identified *L. sceleratus* as a new host for the isopod *E. raynaudii* and therefore was the first report for this isopod species in a tetraodontid fish.

## Conclusions

This study revealed that *L. sceleratus* in the eastern Mediterranean is considered an important host for the low host specificity isopod parasites, providing the negative impact on the affected fish.

## Recommendations

Further studies on the parasitic infestation of the invasive fish species are required and included different localities in the Mediterranean Sea to evaluate the prevalence and impact of the parasites in the long run. The governments of countries that suffer from the spread of the tetraodontid fish on their coasts should ban the fishing and selling these fishes and set laws that contribute the elimination of it and reduce their rates of reproduction.

## Data Availability

The datasets generated or analyzed during this current study are available in this research article and the GENE BANK repository (accession numbers: ON599340.1, OK316895.1).

## Declarations

### Animal handling and use

The work was done on freshly dead animals as the samples were collected alive by fishing net operation then normally died within 1–3 min and immediately investigated. No approval of research ethics committee was required.
